# Models of care and delivery

**DOI:** 10.7448/IAS.17.4.19494

**Published:** 2014-11-02

**Authors:** Jens Lundgren

**Affiliations:** Copenhagen HIV Programme, University of Copenhagen, Copenhagen, Denmark

## Abstract

Marked regional differences in HIV-related clinical outcomes exist across Europe. Models of outpatient HIV care, including HIV testing, linkage and retention for positive persons, also differ across the continent, including examples of sub-optimal care. Even in settings with reasonably good outcomes, existing models are scrutinized for simplification and/or reduced cost. Outpatient HIV care models across Europe may be centralized to specialized clinics only, primarily handled by general practitioners (GP), or a mixture of the two, depending on the setting. Key factors explaining this diversity include differences in health policy, health insurance structures, case load and the prevalence of HIV-related morbidity. In clinical stable populations, the current trend is to gradually extend intervals between HIV-specific visits in a shared care model with GPs. A similar shared-model approach with community clinics for injecting drug-dependent persons is also being implemented. Shared care models require oversight to ensure that primary responsibility is defined for the persons overall health situation, for screening of co-morbidities, defining indication to treat comorbidities, prescription of non-HIV medicines, etc. Intelligent bioinformatics platforms (i.e. generation of alerts if course of care deviates from a prior defined normality) are being developed to assist in providing this oversight and to provide measure of quality. Although consensus exists to assess basic quality indicators of care, a comprehensive set of harmonized indicators are urgently needed to define best practise standards via benchmarking. Such a tool will be central to guide ongoing discussions on restructuring of models, as quality of care should not be compromised in this process.

